# The model medical degree programme “human medicine” in Oldenburg – the European Medical School Oldenburg-Groningen

**DOI:** 10.3205/zma001259

**Published:** 2019-10-15

**Authors:** Kirsten Gehlhar

**Affiliations:** 1 Carl von Ossietzky University of Oldenburg, Faculty VI - Medicine and Health Sciences, European Medical School Oldenburg - Groningen, Oldenburg, Germany

**Keywords:** model medical education programme, academic reform, curriculum development, faculty development, medical studies, outpatient care, scientific research, internationality

## Abstract

In the summer of 2012 the School of Medicine and Health Sciences at the Carl von Ossietzky University of Oldenburg became the first new medical faculty to be founded in Germany in more than 20 years. The faculty was established within the framework of the European Medical School Oldenburg Groningen, a cooperation project between the University of Oldenburg and the University of Groningen. In addition to the University of Groningen and its faculty of medical sciences (Universitair Medisch Centrum Groningen – UMCG), four hospitals in Oldenburg are involved in the programme as cooperation partners, as well as a network of general practitioner practices that provide training and academic teaching hospitals across northwest Germany. The programme itself is a model medical degree programme with a modular structure, a highly integrative approach and an early and consistent focus on practical skills and patient-centredness. In addition to the early introduction to outpatient care in the first years of study, longitudinal pathways and a strong focus on research with early integration of scientific activities into medical studies are the defining characteristics of this programme.

The two faculties in Oldenburg and Groningen coordinated their respective curriculums during the founding phase and recognise each other's study modules as equivalent to their own. This has created the preconditions for students from Oldenburg to obtain Dutch qualifications (Bachelor of Human Life Sciences and/or Master of Science in Medicine) in addition to the German "Staatsexamen" (the state examination in medicine) under certain circumstances. Irrespective of whether they intend to obtain these qualifications, all students from Oldenburg must spend at least a year studying at the partner university in Groningen. In exchange, up to 40 students from Groningen have the option to complete part of their studies in Oldenburg.

## Zusammenfassung

Im Sommer 2012 wurde mit der Fakultät für Medizin und Gesundheitswissenschaften an der Carl von Ossietzky Universität Oldenburg zum ersten Mal seit über 20 Jahren wieder eine medizinische Fakultät in Deutschland gegründet. Diese Fakultät entstand im Rahmen der sog. European Medical School Oldenburg Groningen, einem Kooperationsprojekt mit der Rijksuniversiteit Groningen. Neben der Rijksuniversiteit Groningen mit ihrer universitätsmedizinischen Einrichtung des UMCG (Universitair Medisch Centrum Groningen) sind in Oldenburg vier Krankenhäuser als Kooperationspartner beteiligt. Dazu kommen allgemeinärztliche Hospitationspraxen und akademische Lehrkrankenhäuser im gesamten Nordwesten.

Der Studiengang selbst ist ein modularisierter Modellstudiengang mit stark ausgeprägter Integration und früher und konsequenter Praxis- und Patientenorientierung. Neben der Einführung der ambulanten Medizin in die ersten Studienjahre sind longitudinale Pfade und insbesondere der Forschungsbezug mit früher Einbindung der Wissenschaftlichkeit in das Studium Kennzeichen dieses Studiengangs.

Die beiden Fakultäten in Oldenburg und Groningen haben in der Gründungsphase ihre Curricula aufeinander abgestimmt und erkennen gegenseitig Studienmodule als gleichwertig an. Dadurch wurden Voraussetzungen geschaffen, dass Studierende aus Oldenburg unter bestimmten Voraussetzungen neben dem deutschen Staatsexamen auch niederländische Abschlüsse (Bachelor of Human Life Sciences und/oder Master of Science in Geneeskunde) erwerben konnten. Unabhängig von diesen Abschlüssen müssen alle Studierenden aus Oldenburg mindestens ein Jahr lang an der Partneruniversität in Groningen studieren. Gleichzeitig können bis zu 40 Studierende aus Groningen einen Teil ihres Studiums in Oldenburg verbringen.

## Introduction

### The founding phase 

The model medical degree programme Human Medicine is among the newest medical degree programmes in Germany. After the establishment of the Faculty of Medicine and Health Sciences in 2012 the first students were admitted to the programme in the winter semester 2012/2013. However, the idea of setting up a degree programme in human medicine in Oldenburg originated long before these developments. When the Carl von Ossietzky University of Oldenburg (UOL) was first established in 1969, and again in 1980, plans to set up a medical faculty were discussed but never implemented. After almost ten years of intensive preparation by a small working group made up of members of the participating hospitals and universities, the plans for the medical faculty were finally realized. The idea of integrating the University of Groningen into the project was first discussed in 2004/2005, when the Dutch university was trying to recruit the Klinikum Oldenburg as a teaching hospital for its medical faculty. The initial concept envisaged a two-cycle degree programme in accordance with the Bologna guidelines and based on Groningen's internationally recognised, competence-oriented Bachelor's and Master's degree model. This would have been the first medical degree programme of its kind in Germany. The Federal Ministry of Health, however, rejected the first proposal for this concept initially, mainly on the grounds that the Master's degree was incompatible with Germany's Medical Licensing Regulations for Physicians (ÄAppO). The discussions continued and it was decided that a modified version involving intense cooperation with Groningen and incorporating the founding of the European Medical School Oldenburg-Groningen (EMS-OG) would be adopted. This concept foresaw separate Bachelor phases in Oldenburg and Groningen and a joint Master's phase with an exchange of students between the two locations and two separate Master's degrees awarded by Groningen and Oldenburg. The Master of Science in Medicine conferred by Groningen would also qualify graduates to work in Germany on the basis of the principle of mutual recognition of European professional qualifications. A legal expert report commissioned by the German Council of Science and Humanities (WR) supported this concept and ensured that it fulfilled the constitutional legal requirements [1].

**Figure 1:**
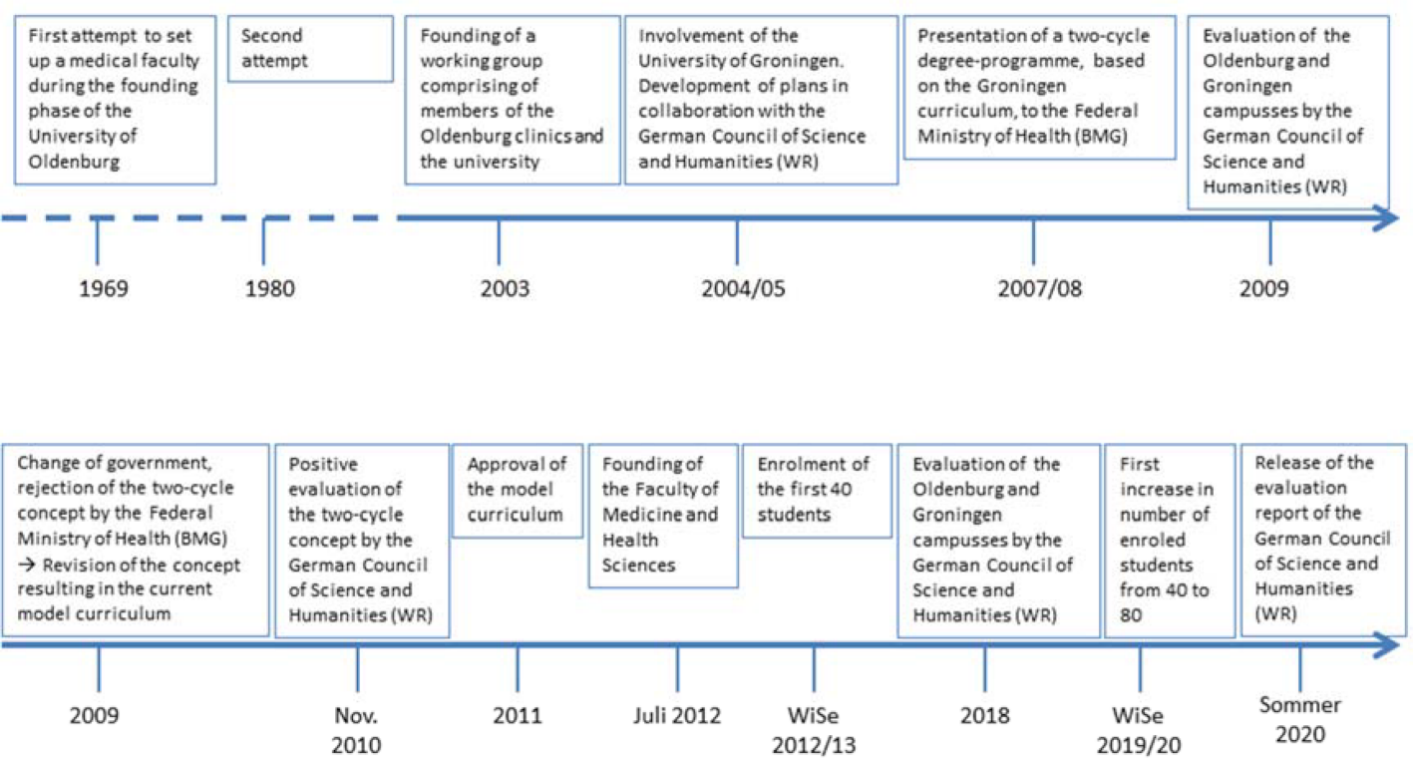
Timeline for the founding phase of Faculty VI Medicine and Health Sciences at the Carl von Ossietzky University of Oldenburg

In October 2009 the medical committee of the WR carried out an inspection in Oldenburg and Groningen and began to compile an advisory opinion [2]. In the view of the WR, one of the main merits of the Oldenburg concept was that it incorporated many elements of the Bologna guidelines, which in Germany in particular [3], but also in other countries, were the subject of criticism in connection with medical education [4]. After a change of government that resulted in a redistribution of posts at the Federal Ministry of Health, the ministry insisted on Oldenburg conferring the state examination qualification (“Staatsexamenabschluss”) – primarily due to the fact that according to the Bologna criteria the Bachelor's degree qualifies graduates for entry into a profession [4], [5]. A specific occupation for holders of a Bachelor's degree in medicine does not currently exist either in Germany or in other countries where Bachelor's degrees are awarded in medical programmes. The Oldenburg University had envisaged the possibility of Bachelor graduates moving on to Master's degree programmes in areas related to medicine, for example the life sciences (e.g. biology) or other health-related programmes. There were never any plans for Bachelor graduates to work as medical professionals [6], [7], [8]. In the end the whole concept was once again revised and the result was a model medical degree programme that conformed to the criteria set out in Paragraph 41 of the ÄAppO (Ärztliche Approbationsordnung – the German Medical Licensing Regulations for Physicians) [https://www.gesetze-im-internet.de/_appro_2002/BJNR240500002.html]. The programme was to adhere as closely as possible to the successful Groningen concept and the current Groningen Curriculum G 2010 [9], but at the same time had to fulfil all the requirements of the ÄAppO. Consequently, the revision was undertaken in close cooperation with the partner university in Groningen. The model medical degree programme Human Medicine was finally approved by state ministry of Science and Culture at the start of 2012, the medical faculty was established in the same year, and the first 40 students were able to commence their studies in the winter semester 2012/2013. Figure 1 (Fig. 1) provides an overview of the founding period and the sequence of events. Four Oldenburg hospitals (the Evangelisches Krankenhaus, the Karl-Jaspers-Klinik, the Klinikum Oldenburg, and the PIUS-Hospital) participate in the implementation of the model medical degree programme. They work together with the Carl von Ossietzky University of Oldenburg on the basis of a framework contract model and together comprise the University's medical campus. 

What remains of the original concept which foresaw a comprehensive cross-border collaboration is an obligatory one year of studies in Groningen for Oldenburg students and the option to earn a Bachelor's or Master's degree in Groningen under certain conditions. In exchange, each year up to 40 Groningen students have the possibility to do a year of the practical training in the Master's phase of their studies at one of the Oldenburg hospitals. The Oldenburg curriculum also still reflects the Bachelor's and Master's concept in its clear division of medical studies into a first and second phase, each of three years' duration. Oldenburg's curriculum also reflects the design of the Groningen curriculum [9] in that the academic year is 40 weeks long – as in Groningen.

The University's medical faculty has now reached the end of its seven-year founding and test phase at the conclusion of which another evaluation in autumn 2018 by the German Council of Science and Humanities was foreseen. Its recommendations to the State of Lower Saxony are expected for the summer of 2019.

#### Organisation and key structural components

In the founding phase of the EMS a number of working groups were set up to work on the planning and implementation of the various sections of the curriculum and the longitudinal pathways. To facilitate the collection and integration of information, all working groups include members of staff from the Deanery of Academic Affairs. Care was taken to include representatives from each clinical facility and from the natural science institutes as well as student representatives in the working groups. In the first years after the faculty's establishment (2011-2015) representatives from Groningen were also in the working groups. 

All the results from the working groups were passed on to the superordinate group, the Working Group Teaching (“AG Lehre”), which in turn is composed of representatives from all the working groups and from the student body. The chairman of the Working Group Teaching is the Dean of Academic Affairs. Minor adjustments to the curriculum were discussed either directly with those responsible for teaching or at the regular meetings of the working groups. 

The Working Group Teaching dealt with administrative matters and innovations in teaching and the study programme. It discussed and approved measures and then passed them on as recommendations to the Study Commission. Under Lower Saxony's Higher Education Act the Study Commission is the body that makes recommendations on all matters related to studies and teaching that are discussed in the Faculty Council. It has an equal number of members from each of the two programmes based at the faculty (Neurocognitive Psychology and Human Medicine), comprising two representatives of the teaching staff, two representatives of the research staff and four students.

The overall management of the curriculum is primarily in the hands of the Deanery of Academic Affairs. It is here that the first drafts for each module are drawn up, annual plans prepared, innovations developed, suggestions from academic staff discussed, and ideas and changes implemented. Academic staff and module coordinators, usually from the dominant (clinical) subject in each module, are in charge of organising instruction, assigning lecturers to courses, revising books for the modules, keeping the literature up to date and helping with the design of exams.

The didactic competence of the lecturers in their particular subject is guaranteed by the broad range of higher education didactics courses on offer at the University of Oldenburg, as well as courses offered within the medical faculty. Together with the Universities of Bremen and Osnabrück and the Technical University of Braunschweig's Competence Centre for Higher Education Didactics, the University of Oldenburg offers the certification programme Higher Education Didactics [https://uol.de/lehre/hochschuldidaktik/zertifikat/], which in compliance with the current standards encompasses 200 training sessions.

In addition, separate workshops on different topics take place within the framework of the *Hochschuldidaktik kompakt* series as well as outside of it. These workshops are open to all members of the teaching staff. The faculty's medicine didactics department also offers its own workshops [https://uol.de/medizindidaktik]. These internal workshops are held on a regular basis specifically for teaching staff in the model medical degree programme. 

## Methodology

### Content

The University of Oldenburg's model medical degree programme Human Medicine applies innovative methods in the education of medical students and aims to meet the demand for the qualified training of physicians for the region and offer students an excellent scientific education. The competence-oriented training focuses on patient-centeredness, doctor-patient communication, vertical and horizontal integration, teaching of scientific practice and the strengthening of general medicine. 

#### Years 1 to 3 of study

**Figure 2:**
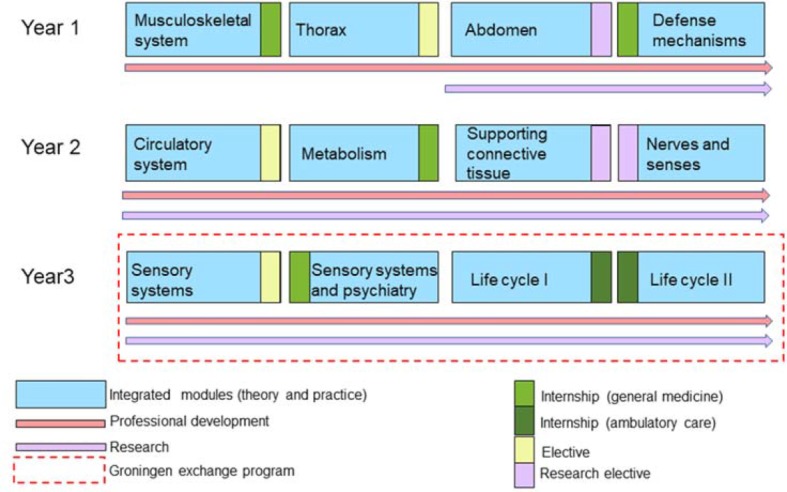
Curricular structure of the first three years of study

The first three years of study are each divided into four 10-week modules that build on each other. In these modules students are taught preclinical science and clinical content in a consistently interdisciplinary and integrative approach (see figure 2 (Fig. 2)). The first year of studies focuses on the acquisition of basic knowledge; the goal is for students to gain an understanding of the normal functions of a healthy body. From the beginning the basic sciences and clinical practice are taught in an integrative approach, so for example in the first semester the clinical foundations of orthopaedics are supplemented by a course in basic orthopaedic examination, instruction in the corresponding fundamentals of anatomy and physiology of the musculoskeletal system, and an “anatomy-in-vivo” course. Parallel to this, content from physics provides a basic understanding of the forces that act upon the musculoskeletal system. 

The second and third year of study follow up on the basic knowledge taught in the first year. The modules are clinically oriented but – building on the basic knowledge of physiology acquired in the first year – a stronger focus is placed on diseases and their treatment. With this approach, in their first three years of medical education students learn not only the content relevant for their Basic Medical Examination (“Physikum”) but also acquire a substantial proportion of the basic knowledge required in the clinical subjects. Students conclude the first three years with the equivalent of the first part of the Medical Examination (M1). 

A special feature of the Oldenburg medical degree programme is that it incorporates a series of longitudinal pathways that run parallel to the themed modules across the entire curriculum. These are:

- Communication
- Clinical examination
- Scientific work (longitudinal research curriculum = LRC)
- Professional development

#### Years 4 to 6 of study

In the fourth year of study the practical component substantially increases and the building blocks of the first three years (clinical knowledge, communication, examination) are merged. Students complete four five-week block internships at hospitals; these practical training phases are each preceded by five weeks of preparatory instruction at a clinical training centre. In this fourth year students also deepen their knowledge in the pathway “Communication” during four so-called “consultation weeks” in which course content is horizontally combined with clinical content.

In the fifth year of their medical training students spend 4 to 5 four-week blocks in hospital departments (three compulsory internship blocks in accordance with the Medical Licensing Regulations, one in an elective clinical subject and an optional internship block) and write the so-called major research paper (“große Forschungsarbeit”) within 20 weeks. Students are free to decide in which order they wish to complete these phases of their studies. 

**Figure 3:**
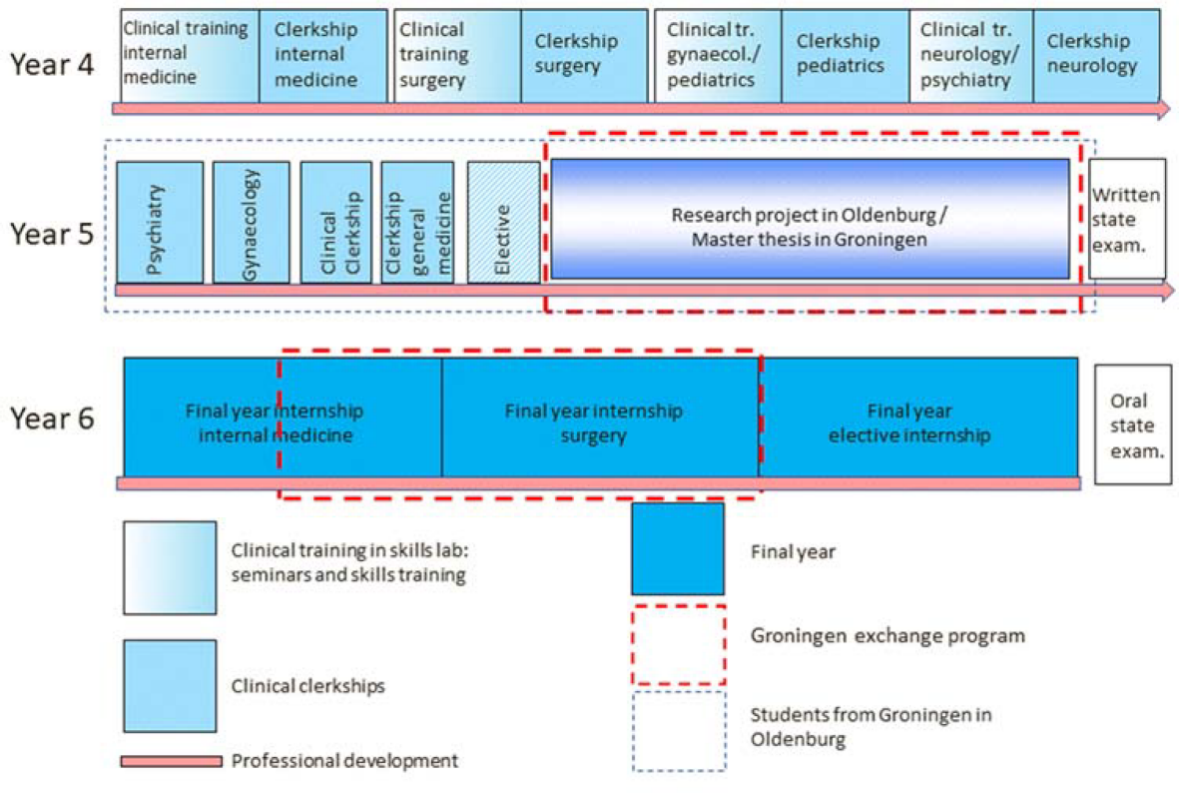
Curricular structure of the final three years of study

After the written second part of the state examination students complete a practical year divided into three four-month segments (“Tertiale”) and conclude their studies with the oral part of the second state examination (see figure 3 (Fig. 3)). 

#### Practical Training in general medicine and outpatient care

In addition to formal, structured courses, students spend a total of six weeks completing medical internships – known as “hospitations” – at outpatient care facilities such as private or group practices, four of which must be general medicine practices. The first hospitation week already takes place in the 10th week of study. The goal is to implement the knowledge and skills acquired in the preceding modules and practice and deepen them in a supervised setting. In order to achieve this, specific topics are set in the hospitations, prepared at campus events, and put into practice through the formulation of concrete objectives and the assignment of tasks. A logbook is compiled for each hospitation. Students are trained exclusively at medical practices across northwest Lower Saxony that have received special instruction in offering a particular type of hospitation. Once they have completed the general medicine **hospitations students complete another a two-week hospitation in the third study year at a specialised outpatient practice** of their choosing.

## The longitudinal pathways

### Communication

From the first module students learn about medical communication and receive systematic training to improve their skills in this area in the “Communication” course series. The curriculum encompasses approximately 150 hours. In the fourth year of study they deepen their knowledge and skills in this area during four “consultation weeks” in which practice with simulated patients is horizontally combined with clinical content. Students are examined on this content in each of the six OSCE Examinations (objective clinical examinations, [10]) they take in the first three years of study. 

#### Clinical examination 

The “Clinical and Practical Skills” pathway offers practical examination courses in each module which are tailored to the weekly or module topics. In years 1-4, a total of 75 compulsory courses (equivalent to 160 hours) are offered. Additional, optional, in-depth courses are held in years 5 and 6. The students' clinical and practical skills are tested in the six OSCEs [10] which take place in years 1-3 and in a MiniCEX [11] assessment in year 4.

#### Science and research in the programme

A special feature of the Human Medicine model degree programme is the early and longitudinal involvement of students in scientific activities and research. Their scientific education begins in the first year of study and, combined with instruction in the intersection area of epidemiology and biometrics, continues throughout the programme as a longitudinal element (= **Longitudinal Research Curriculum, LRC**), until at least the start of the Practical Year (“Praktisches Jahr” – the final year of studies). In the first three study years the LRC focuses on teaching scientific methods and the scientific foundations of medicine and providing an introduction to independent research. In the first year of study students are introduced to research by being assigned to small groups and given a research project to prepare and complete within one week. 

Their work in the project is closely supervised by a mentor. To complete the project and earn the credits the groups design one or several posters in English as well as an abstract for each poster, also in English. In the second and third years, parallel to their studies students prepare a research project independently and write a so-called “Small Research Paper” (“kleine Forschungsarbeit”). In the winter semester 2018/2019 this concept will be replaced by a structured data analysis project. In year 4 the training continues in the *Journal Clubs*. In these clubs, students read scientific texts and analyse and interpret the results. In the fifth year of study students complete the “Big Research Paper” (over a 20-week period). In this project students can continue their activities from the first three years or conduct research in a new area. They also have the option to prepare their doctorate in the context of this project or to use the project as the foundation for their doctorate. The model degree programme thus fulfils the requirements of the German Council of Science and Humanities (WR) [12], which in 2014 spoke out clearly in favour of a stronger focus on scientific competences in medical education and proposed that the acquisition of these competences should be a central, ideally incremental component of the students' education. This was reaffirmed in the Council's recommendations [13] for the Master Plan Medical Education 2020 (“Masterplan Medizinstudium 2020” [14]). 

#### Professional development

In the longitudinal pathway **Professional Development** [15] which spans the entire six years of study, students receive guidance for their professional development. Practical experiences are discussed and analysed, and summarised in a Portfolio throughout the study program [16]. In addition, a variety of interdisciplinary topics which overlap with linguistics, the humanities, the social sciences or cultural studies are discussed.

#### Didactics/teaching formats 

Taking the Groningen degree programme G2010 [9] as its model, Oldenburg established its own model medical degree programme characterised by an unusually early and strong integration of subjects and a focus on practical training and patient-centredness. Consequently, in the first three years of study as well as conventional lectures and seminars the programme incorporates a number of alternative teaching formats aimed at achieving this goal: 

#### Patient-centred teaching formats

From the first day of study and thoughout the first three years of study students attend weekly **Patient Seminars** (“**Patientenkollegs**”). Tutors bring outpatients to these seminars with complaints that serve as an introduction to the week's topic. The purpose of this early integration of patients into the course is that students become accustomed to talking to patients and develop their skills in taking medical histories and clinical reasoning [17]. These seminars are not primarily aimed at acquiring clinical knowledge on the basis of patient contact.

From the second year of studies onwards a general practitioner and a specialist physician discuss and resolve a patient case together with students in weekly **Problem-Solving Seminars**. The goal of this teaching format is that students learn and practice clinical reasoning and differential diagnostics in clinical and outpatient care situations on the basis of authentic patient cases and with the active participation of the students, in order to increase the value and effectiveness of the learning situation [18], [19], [20]. The path to the solution starts with a structured anamnesis with emphasis on the relevant fields, a clinical examination and technical examinations including laboratory tests. After each step students make suggestions regarding a diagnosis, eliminate other diagnoses and propose further tests. The lecturers scrutinise the suggestions and differential diagnoses, answer questions and discuss the advantages of the suggestions for solving the problem in a step-by-step process. The targeted diagnosis is embedded in the curriculum and tailored to the week's topic. 

The** Interactive Seminar** in the fourth year of study is a continuation of the “Problem-Solving Seminars” of years 2 and 3. Prior to the seminar students are given a paper-based patient case to work on in groups of 4-6 students. The case is then discussed in the seminar with the active participation of the students [20]. Depending on the topic, students are asked to prepare and present specific case-related topics (specific treatment modes, current scientific results or the like). 

In the **Problem-based-learning** (PBL) tutorials which take place in the first three study years, small groups of 8-10 students work together on a patient case that is relevant to the week's topic. A strong emphasis is placed on formulating the learning objectives that are also integrated into the OSCE exams in the first two years of study. In these assessments students do a presentation on a PBL learning objective from the previous module at an OSCE station in the form of a short presentation. Both content and presentation skills are assessed. The purpose of this method of assessment is not to test whether a student can reproduce the concept or procedure of PBL, as is the case with Triple Jump Examinations, for example [21], [22]. Instead the focus is on the last step of the PBL in which the students of a PBL group present their learning objectives and perform an aspect of the “Scholar” role from the CANMEDs framework [23], which can also be found in the National Competence-Based Learning Objective Catalogue for Medicine (“Nationalen Kompetenzbasierten Lernzielkatalog Medizin” – NKLM [http://www.nklm.de]) in the chapter “The Physician as a Scholar” (“Die Ärztin/der Arzt als Gelehrte/r”, see NKLM: Lernziel 6.3). The didactic concept on which the POL tutorials are based is continually adjusted to match the students' educational level as they progress through their studies. Whereas during the first year of study Maastricht University's classic seven-step approach [24] is applied, in the second year of study students are given additional tasks such as drafting treatment plans or conducting a pre-operation discussion corresponding to a specific PBL scenario. In the third year of study tutors receive comprehensive guidelines for the PBL scenarios including detailed explanations of diagnostic methods, differential diagnoses and treatment, whereas students are given only a brief introduction to a case prior to the session, meaning that in most cases they prepare in advance using textbooks (based on [25]). During the session students take the patient's medical history (tutors prepare in advance to play the patient role) and use it to formulate a working diagnosis. This serves as the starting point for further learning objectives, reflection on potential treatments and simulated conversations with patients. 

#### Evaluation

Students acquire a state-recognised equivalent of Part One of the National Medical Licensing Exam (“Ärztliche Prüfung – M1”) within six semesters of the standard time to degree. The written part comprises all the questions from the written module exams taken in the first three study years (approx. 100 questions at the end of each of the 12 modules), which are taken from the subjects listed in Paragraph 22 of the Medical Licensing Regulations for Physicians (ÄAppO). The oral part is calculated on the basis of the average scores from all six OSCE exams, which from years 1-3 are held at the end of each semester, with six stations for each exam. 

Since all the module examinations of the first three years of study comprise a combination of pre-clinical and clinical subjects due to the interdisciplinary and integrative structure of the degree programme, all the questions in the written exams are taken from the subjects stipulated in Paragraphs 22 or 27 of the Medical Licensing Regulations for Physicians, and once they have passed a module exam students receive credits on individual “subject accounts”. The questions are primarily based on the learning objectives for individual courses as defined in the module handbooks. After the third year of study only the accounts for the subjects and interdisciplinary areas listed in Paragraph 27 of the Medical Licensing Regulations (ÄAppO) are filled. In addition to summative evaluations students are obliged to take the formative Progress Test Medicine of the Charité Berlin each semester [26].

The faculty also uses many other written and oral and oral/practical assessment methods.

Providing students with feedback is a central component of the model degree programme. In all (block) internships, hospitations and other practical training formats, supervisors complete a standardised feedback form for each student which in the first three years of study forms part of their professional development portfolio and in years 4 and 5 part of the logbooks. Students reflect on the feedback they receive in written form as well as in discussions with the tutors who guide their professional development.

## Results

A first comprehensive evaluation of the programme was carried out from 02.05 - 05.05.2016 by the 11^th^ cohort of the Master of Medical Education degree programme in Heidelberg, which evaluated the teaching situation within the framework of its final module. In preparation for the evaluation the faculty drafted a self-assessment report structured according to the criteria of the World Federation for Medical Education (WFME) – Global Standards for Quality Improvement [27]:

1. Mission and outcomes
2. Educational programme
3. Assessment of students
4. Students
5. Academic staff /faculty
6. Educational resources
7. Programme evaluation
8. Governance and administration
9. Continuous renewal (not applicable for Oldenburg at the time of evaluation)

Further questions beyond the scope of the WFME criteria as well as location-specific questions posed by the evaluation group were also answered in written form in advance.

During the week of the evaluation group's visit numerous interviews took place with all the involved parties (university management, coordinators, administration, academic staff, students) in which the questions of the evaluators were discussed very openly. A visit was also made to the partner university in Groningen. The results of the three final reports in the areas “educational programme” and “assessment of students” can be summarised as follows: the evaluation committee praised in particular the early integration of clinical knowledge and practical skills, the focus on early acquisition of skills in interacting with patients, the longitudinal pathways, the successful modular structure incorporating a high proportion of work in small groups, and the early emphasis on the development of research skills. It also gave the cross-border cooperation combined with a regional focus (the network of medical practices in the region involved in student training) a positive assessment. Points of criticism were that the curriculum needed to be streamlined due to lacking flexibility in terms of time management, that there are not enough optional courses, and that because of the workload there is no possibility to earn a doctorate parallel to completing the programme. 

In the area of assessment of students, the ultra-modern electronic assessment system with its diverse assessment methods, fast rhythm of assessment and two-tier review system (form and content) were commended. The use of the compulsory Progress Test as a benchmark was also praised, as was the largely successful coordination of academic staff, learning objectives, examinations and assessment methods. The evaluators also voiced criticism in this area, pointing out that the assessment methods require considerable administrative effort, that in certain areas academic staff and module coordinators are not sufficiently involved in determining the content of examinations, and that there is a lack of synchronisation between teaching and assessment in certain areas. 

Faculty VI Medicine and Health Sciences was evaluated by the German Council of Science and Humanities (WR) according to plan in October 2018. The faculty compiled a self-assessment report, which was submitted to the WR via the Ministry for Science and Culture of the State of Lower Saxony (MWK) in June 2018. The evaluation panel of the WR visited the medical faculty and the hospitals in Oldenburg on 25 October and then Groningen on 26 October to form an opinion of the faculty and the two locations. During meetings all the parties involved with Oldenburg's school of medicine, including the presidential chair, the faculty and hospitals and the Lower Saxony Ministry for Science and Culture answered questions posed by the WR. The recommendations of the Council are expected for mid-2019. In accordance with Lower Saxony's Higher Education Act (NHG [http://www.schure.de/22210/nhg.htm]) these recommendations are to form the basis for the state's decision regarding the further development of the faculty of medicine and the degree programme. 

## Discussion/Outlook

The situation of the faculty improved considerably in the winter semester 2018/2019. The WR's visit ended with positive signals regarding the progress made to date in Oldenburg. In addition, the State of Lower Saxony, encouraged by the objective of increasing the number of study places by 200 (see Section 7b [28]) stipulated in the current state government's coalition agreement, brought forward an amendment of its Higher Education Act to 18 December 2018. With effect from 01.01.2019 the School of Medicine's annual student intake capacity was doubled from 40 to 80 students as of the winter semester 2019/20. At the same time, in order to improve the collaboration between the University and the participating hospitals during this expansion phase, the right to a say of mid-level medical staff at the hospitals participating in the teaching and research was strengthened (NHG § 63i (3)). With these steps the faculty moved from the test phase to the expansion phase. Even before the WR's visit the University had already agreed with the State of Lower Saxony that the Faculty would increase its annual intake capacity to 200 students in the long term. The State of Lower Saxony has approved a progressive increase from 2020 on in the funding allocated to the faculty, commensurate to the rising student numbers, in order to cover the growing tasks in teaching and research and provide resources for the necessary infrastructure/building measures. 

The decision to increase the student intake to 80 earlier than planned, in the winter semester 2019/2020, also has major implications for the cooperation with the partner university in Groningen. The latter has so far made both facilities and materials available for practical courses in anatomy and also reserved sufficient capacities to be able to offer all Oldenburg students the option of spending one year of their studies in Groningen. These capacities were limited to 40 students per year group, and were non-extendable. During a visit by the Minister of Science for Lower Saxony Björn Thümler to Groningen in June 2018, an agreement was reached with the president of the University of Groningen to expand the cooperation between the two universities. This also includes increasing Groningen's capacities for Oldenburg students to 80 students per year, initially for a limited period of time. The details of how this is to be implemented are currently the subject of intense dialogue between the two faculties. 

Groningen's limit of 80 exchange students means that as the number of medical students at Oldenburg continues to grow in accordance with the terms of the agreement with the State of Lower Saxony, Groningen can no longer be integrated into the programme to the extent that it has been so far. Consequently, a new concept is currently being developed to take account of the long-term plans for higher intake figures in Oldenburg. 

Quite apart from Groningen's limited capacities, the teaching capacities in Oldenburg are not yet sufficient to cope with a considerable rise in the number of medical students. The curriculum is tailored to a small number of students (see above). Although the Groningen curriculum [9], much of which was adopted in 2011, was implemented for more than 400 students per year there, the conditions in Groningen cannot be replicated without considerable adjustments in a small medical faculty like Oldenburg's. In the meantime, Groningen University has also revised its medical curriculum [29] and among other changes considerably reduced the proportion of attendance-based lectures and seminars. In Oldenburg the curriculum must now be adapted to larger groups in a faculty-wide coordination process. This process began at the start of 2019. It also offers the opportunity to incorporate ideas for improvements that have emerged in the seven years since the programme's launch. The reform has two main objectives: to streamline the curriculum (a reduction in the total number of courses offered, currently encompassing well over 5,500 hours). 

The process will aim to identify and eliminate redundancies and streamline the modules without endangering the existing integrative components. At the same time the patient-centred and special teaching formats as well as the longitudinal pathways and the focus on science and research described in this paper, which follow up on many of the reforms in medical education proposed by the WR [12] and the Masterplan 2020 [14] and recommended by the WR [13], are to be maintained, but if necessary their frequency will be reduced. The clinical training capacities are also virtually exhausted at present. In this area too, a new concept that doesn't overstretch the existing resources and structures is required. This concept will involve changes to the curriculum and also structural modifications to the procedures in the hospitals as well as the recruitment of further hospitals if necessary.

At the same time more professors will also be appointed to teach the subjects listed in the Medical Licensing Regulations that are not yet covered in the programme. In addition, new staff must be recruited for major subjects that require substantial teaching resources. Another top priority is the rapid construction and expansion of the required infrastructure for teaching and research. This includes the establishment of an anatomy department at the medical faculty. The Ministry for Science and Culture of the State of Lower Saxony has signalled its willingness to provide funding for a first building for teaching and research, the planning of which began this year.

## Competing interests

The author declares that she has no competing interests.
